# Retraction: Hybrid deep learning and feature selection approach for autism detection from rs-fMRI data

**DOI:** 10.1371/journal.pone.0354652

**Published:** 2026-07-27

**Authors:** The PLOS One Editors

After this article [1] was published, concerns were raised regarding:

- Compliance with the PLOS Authorship policy.
- High similarity with a study and results previously reported in [2] that was not cited or discussed in [1].
- Highly similar results in Tables 1 and 2 of [1] and [2] representing different brain atlases derived from the same ABIDE-1 dataset and representing different algorithmic methods.
- References 26, 28 and 50 were retracted while [1] was under review.

The fourth author acknowledged that [2] should have been cited in [1] and disclosed at submission as a similar publication. In relation to the highly similar results representing different atlases and algorithms in the two articles, the fourth author stated that the datasets are mislabeled in [1], clarifying that Tables 1-3 in [1] present the AAL, EZ and CC datasets, respectively. They claimed that different algorithms applied to the same dataset and atlas can produce highly similar results and that the results from the two articles diverge at the 6–8th decimal place, which is not apparent in the published tables as the data are rounded to four decimal places. The underlying data files provided were not sufficient to resolve the concerns, and the *PLOS One* Editors do not consider this matter fully resolved.

In light of the concerns regarding the reliability of the reported results and the article’s compliance with PLOS policies, the *PLOS One* Editors retract this article.

MAE, AAE, MGK, AD, and IAF did not agree with the retraction. NM, SMA, IN, and MAAB either did not respond directly or could not be reached.
